# Mechano-induced cell metabolism disrupts the oxidative stress homeostasis of SAOS-2 osteosarcoma cells

**DOI:** 10.3389/fmolb.2023.1297826

**Published:** 2024-04-25

**Authors:** Giuseppina Fanelli, Giulia Alloisio, Veronica Lelli, Stefano Marini, Sara Rinalducci, Magda Gioia

**Affiliations:** ^1^ Department of Ecological and Biological Sciences (DEB), University of Tuscia, Viterbo, Italy; ^2^ Department of Clinical Sciences and Translational Medicine, University of Rome “Tor Vergata”, Rome, Italy

**Keywords:** metabolomics (LC-MS), osteosarcoma, mechanobiology, oxidative stress response, cyclic stretch

## Abstract

There has been an increasing focus on cancer mechanobiology, determining the underlying-induced changes to unlock new avenues in the modulation of cell malignancy. Our study used LC-MS untargeted metabolomic approaches and real-time polymerase chain reaction (PCR) to characterize the molecular changes induced by a specific moderate uniaxial stretch regimen (i.e., 24 h-1 Hz, cyclic stretch 0,5% elongation) on SAOS-2 osteosarcoma cells. Differential metabolic pathway analysis revealed that the mechanical stimulation induces a downregulation of both glycolysis and the tricarboxylic acid (TCA) cycle. At the same time, the amino acid metabolism was found to be dysregulated, with the mechanical stimulation enhancing glutaminolysis and reducing the methionine cycle. Our findings showed that cell metabolism and oxidative defense are tightly intertwined in mechanically stimulated cells. On the one hand, the mechano-induced disruption of the energy cell metabolism was found correlated with an antioxidant glutathione (GSH) depletion and an accumulation of reactive oxygen species (ROS). On the other hand, we showed that a moderate stretch regimen could disrupt the cytoprotective gene transcription by altering the expression levels of manganese superoxide dismutase (*SOD1*), Sirtuin 1 (*SIRT1*), and NF-E2-related factor 2 (*Nrf2*) genes. Interestingly, the cyclic applied strain could induce a cytotoxic sensitization (to the doxorubicin-induced cell death), suggesting that mechanical signals are integral regulators of cell cytoprotection. Hence, focusing on the mechanosensitive system as a therapeutic approach could potentially result in more effective treatments for osteosarcoma in the future.

## 1 Introduction

The human body is constantly affected by physical forces which change molecular properties determining how cells behave. Over the past 20 years, there has been extensive research on mechanosensing and mechanotransduction, particularly in adherent cells (Michaletti et al., 2017; Mohammed et al., 2019; Alloisio et al., 2021). These processes are now recognized to play vital roles in numerous essential cellular functions, including adhesion and proliferation, mitosis, motility, and apoptosis (Holle and Engler, 2011; Gioia et al., 2018; Alloisio et al., 2021; Harris and Hawkins, 2022). Similarly, cancer cells exert forces to facilitate their spread to other parts of the body while consuming ATP. Recently, there has been an increasing focus on cancer mechanobiology, determining how cells behave to unlock new avenues in the modulation of cell malignancy (Bertero et al., 2019; Park et al., 2020; Hattinger et al., 2021; Talayero and Vicente-Manzanares, 2023). During solid tumor progression, cells undergo mechanical and metabolic changes that sustain migration. However very little is known about how the metabolic state of a cell affects its malignant properties and *vice versa* (Schiliro and Firestein, 2021).

Osteosarcoma (OS) stands as the most prevalent primary bone tumor. It exhibits a bimodal age distribution, with a peak occurrence of 4.2 cases per million in children and young adults, followed by another peak of 4.2 cases per million in older individuals (Harris and Hawkins, 2022). Despite the customary practice of subjecting OS patients to an intense chemotherapy regimen involving multiple drugs both before and after surgery (Lilienthal and Herold, 2020), there has been no notable enhancement in the survival rate for metastatic osteosarcoma for several decades (Harris and Hawkins, 2022). Collaborative endeavors have yielded minimal advancements in the survival prospects of individuals with metastatic osteosarcoma. Consequently, there is an urgent demand for novel treatment approaches for OS.

Cancer cell mechanobiology has been recently explored for OS cells by showing that they modulate their malignancy in response to changes in several extracellular mechanic stimulations (Adamopoulos et al., 2017; Bertero et al., 2019; Müller and Silvan, 2019; Shoaib et al., 2022). Noteworthy, recent studies have identified compelling links between extracellular signals and chemotherapy failure (Yu et al., 2022), envisaging the possibility that extracellular stimulations can regulate cell cytotoxicity. The bone microenvironment has been shown to play a crucial role in the development and progression and tumor relapse of OS since the cell subpopulation of the bulk tumor became resistant to cytotoxic drugs (Hattinger et al., 2021). Hence, given that the majority of chemotherapeutic agents are known to increase the intracellular concentrations of reactive oxygen species (ROS) and can disrupt the redox balance within cancer cells (Yang et al., 2018), it is a plausible hypothesis that mechanical forces contribute to cytotoxic processes in cells. Accordingly, a mechanically-induced morphological alteration has been reported to be associated with an acute cytotoxicity effect on the human breast cancer cell line (Dufrêne et al., 2017). Moreover, several studies have shown that the upregulation of the expression of some mechanosensitive cytoprotective genes such as manganese superoxide dismutase (*SOD1*), Sirtuin 1 (*SIRT1*), NF-E2-related factor 2 (*Nrf2*) and Transcription factor forkhead box protein O1 (*FOXO1*), is a necessary survival adaptation during tumor progression (Maiese et al., 2009; Park et al., 2012; Zhang et al., 2016; Xue et al., 2020; Eleutherio et al., 2021). Mounting evidence has proposed this enabling cells to cope with increased cellular and extracellular redox stress (Kajihara et al., 2006; Lee et al., 2013; Barrera et al., 2021; Kuno et al., 2023).

Commonly, metabolic alterations occurring in most cancer cells are believed to contribute to tumor development and also mediate resistance to chemotherapeutical drugs (Chen et al., 2020). In particular, alterations of cell metabolism in OS, which have been previously reported to be correlated to the grade of OS malignancy (Lamego et al., 2014; Li et al., 2016; Ren et al., 2017), and understanding the mechanism of transformation during metastasis have provided information on treatment and prognosis (Fritsche-Guenther et al., 2020). We have recently selected a mechanical regimen (i.e., 24 h-1 Hz-cyclic stretch 0.5% elongation) able to disrupt the metastatic potential properties (such as cell migration and adhesion) of the SAOS-2 osteosarcoma cell line (Alloisio et al., 2023). The current study intended to identify the mechano-induced alterations of metabolism that sustain the mechano-biological changes of SAOS-2 cells by using an untargeted mass spectrometry approach. In particular, since several mechanical stimuli are reported to increase the production of free radicals such as reactive oxygen and nitrogen species via several mechanisms (Djordjevic et al., 2010; Palomero et al., 2012), our metabolomics analysis was also focussed on the mechano-modulation of the oxidative stress homeostasis. Moreover, a quantitative real-time PCR comparative analysis, between mechanically treated SAOS-2 cells and static control counterpart, was performed on the expression levels of some mechanosensitive cytoprotective genes such as *SOD1*, *SIRT1*, *FOXO1* and *Nrf2* (Pardo et al., 2008; Chen et al., 2021; 2018), which are reported to be involved in the defence against oxidative stress (Kajihara et al., 2006; Lee et al., 2013; Barrera et al., 2021; Kuno et al., 2023). Lastly, we screened whether or not the mechanical-induced metabolic changes could help osteosarcoma SAOS-2 cells to become more sensitive to doxorubicin-induced death.

## 2 Materials and methods

### 2.1 Cell culture

The SAOS-2 human osteosarcoma cell line was sourced from the Biological Bank and Cell Factory-Interlab Cell Line Collection (ICLC) (Accession Number ICLC HTL01001) IRCCS Policlinico San Martino Hospital, IST Genova, Italy. These cells were cultured in Dulbecco’s Modified Eagle’s Medium (4.5 g/L glucose)/Ham F12 (1:1) (Invitrogen, Carlsbad, CA, United States) and supplemented with 10% fetal bovine serum (FBS) (Euroclone s.p.a., Milano, Italy), Penicillin–Streptomycin Solution 100X (Gibco, Life Technologies, Carlsbad, CA, United States), and Amphotericin B 100X (Biowest, Riverside, MO, United States) at 37°C in an atmosphere of 5% CO_2_. The culture medium was refreshed twice a week, and any nonadherent cells were removed during these routine changes.

### 2.2 Mechanical stretch application

A cyclic uniaxial stretch of 0.5% elongation at 1 Hz for 24 h was applied to adherent cells using the MechanoCulture FX device, as previously outlined in Alloisio et al. (2023). Briefly, a deformable silicone well culturing plate from CellScale Biomaterials Testing in Waterloo, ON, Canada, was employed to hallow the culturing system to follow the uniaxial deformation applied. These silicone wells were pre-coated with rat type I collagen from Enzo Life Sciences in Farmingdale, NY, United States, at a concentration of 50 μg/mL in a PBS solution. SAOS-2 cells were seeded at a density of 2 × 10^5^ cells per well, with a culture area comprising 16 wells, each measuring 8 mm × 8 mm. For each dataset, two silicon plates were seeded, and both were exposed to the same experimental conditions. However, only one of the two plates underwent the mechanical stretching, while the other served as a static isometric, unstimulated control. After 24 h of cell seeding, a 0.5% elongation stretch was applied to the cells at a cyclical frequency of 1 Hz for 24 h, with a pattern of 1 h of stretching followed by 3 h of rest. To assess the response of adherent live cells immediately after the mechanical stretch, an in-house developed micro-plate adapter was used to detect fluorescence and spectrometry, employing a TECAN spark microplate reader (Tecan Group, Männedorf, Switzerland) (Alloisio et al., 2023).

### 2.3 Metabolite extraction and UHPLC‒MS analysis

Immediately following the mechanical treatment, the stretched samples and their respective control counterparts were subjected to the following processing steps. Cell Counting: The cell number was determined by counting the cells after trypsinization using an automated cell-counting chipTM on the TECAN sparkR multimode reader, a product of the Tecan Group based in Männedorf, Switzerland. Sample Resuspension: The sample was resuspended by adding 0.15 mL of ice-cold ultra-pure water with a resistance of 18 MΩ to lyse the cells. The tubes containing the samples were rapidly subjected to two temperature shifts: first, they were plunged into dry ice or a circulating bath at −25°C for 0.5 min and then transferred to a water bath at 37°C for another 0.5 min. Chemical Extraction: To each tube, 0.6 mL of −20°C methanol and then 0.45 mL of −20°C chloroform were added. The tubes were mixed every 5 min for a total of 30 min. Subsequently, the solutions were centrifuged for 15 min at 15,000 × g before being stored at −20°C for 2–8 h. Further Centrifugation: The tubes were then centrifuged again at 10,000 ×g for 10 min at 4°C, and the collected supernatants were dried to obtain visible pellets. Sample Re-suspension and LC/MS analysis: Finally, the dried samples were re-suspended in 0.1 mL of a solution containing water and 5% formic acid. These re-suspended samples were then transferred to glass autosampler vials and analyzed by LC/MS as previously reported (Tzounakas et al., 2022).

### 2.4 Metabolomic data processing and statistical analysis

Metabolomic data processing and statistical analysis involved the following steps. Data Export and Pre-processing: Raw data files from replicates were exported as.mzXML files and subsequently processed using MAVEN 8.1. Statistical analysis: Univariate (Volcano plots) and multivariate statistical analyses were performed on the complete metabolomics dataset using MetaboAnalyst 5.0 software. Before analysis, the raw data underwent normalization by median and autoscaling to give greater importance to low-abundance ions while minimizing the amplification of noise. False discovery rate (FDR) was used for controlling multiple testing. Pathway Analysis: Pathway analyses were performed using the web-based tool MetPA (Metabolomic Pathway Analysis), integrated into the MetaboAnalyst platform. Data for metabolites detected in all samples were input into MetPA with annotations based on common chemical names. These accepted metabolites were manually verified using databases like HMDB, KEGG, and PubChem. For pathway analysis a *Homo sapiens* pathway library was utilized; the global test was the chosen pathway enrichment analysis method, and the relative betweenness centrality was the node importance measure for topological analysis. Graphical Representation: Metabolites that displayed significant changes were graphed and statistically analyzed by using Graphpad Prism 5.01 software. The reported data represent the means of three replicates with standard deviation (SD) indicated.

### 2.5 Analysis of intercellular ROS generation on flexible silicone plates

The intercellular ROS formation was evaluated using a DCFDA/H_2_DCFDA- Cellular ROS Assay Kit (ab113851, Abcam, Cambridge, United Kingdom). Cells were seeded on a silicone plate at a density of 400 cells/mm^2^ and cultured in phenol red-free Dulbecco’s Modified Eagle’s Medium (4.5 g/L glucose)/Ham F12 (1:1) (Corning, Manassas,VA, United States) and supplemented with 10% fetal bovine serum (FBS) (Euroclone s.p.a., Milano, Italy), Penicillin–Streptomycin Solution 100X (Gibco, Life Technologies, Carlsbad, CA, United States), and Amphotericin B 100X (Biowest, Riverside, MO, United States) at 37°C in an atmosphere of 5% CO_2_. These cells were cultured for a day to allow cell adhesions. The day after the medium was replaced with phenol red-free medium. Then one plate underwent to 1 Hz cyclic stretch for 24 h, whereas the second plate was left static under the exact condition of the stimulated well. The ROS formation was performed within the two silicone plates according to the manufacturer’s instructions of the DCFDA/H_2_DCFDA - Cellular ROS Assay Kit (ab113851, Abcam Cambridge,United Kingdom). Briefly, the cell medium was discarded, and the attached cells were washed twice with 100 µL of 1X buffer per well. Then cells were incubated with an H_2_DCFDA probe and stained at 37°C with 5% CO_2_ in the dark. Cell medium was discarded, and the attached cells were washed twice with 100 µl of 1X buffer per well. 55 μM MBHP (ter-butyl hydroperoxide) solution was used to develop the fluorogenic reaction. First fluorescence reading was recorded at t(0) using 485 and 535 nm as Excitation and Emission wavelength, respectively. The silicone plates were maintained at 37°C with 5% CO_2_ in the dark and after 2 h of incubation, the fluorescence was detected again by an Infinite^®^200 PRO multi-well plate reader (Tecan Group Ltd., Männedorf, Switzerland). Additionally, a treatment that enhances ROS production was performed. Specifically, both pates underwent 2-h incubation with 8,6 µM doxorubicin holding cells at 37°C with 5% CO_2_ in the dark.

### 2.6 Cytotoxicity assay

The impact of mechanical pre-treatment on cell cytotoxicity induced by doxorubicin was measured by an MTT colorimetric assay (Merk Life Science S.r.l., Milano, Italia). To examine doxorubicin cell cytotoxicity, cells were seeded on silicone plates at 110 cells/mm^2^ density and allowed to adhere for 24 h as previously indicated. Then, cells on the silicone-plate were 24 h-treated with serum-free medium at different concentrations of doxorubicin within the range of 0–10 μM. For the measurement of absorbance after 24 h, each well received 20 μL of MTT solution (5 mg/mL in PBS with Ca^2+^ and Mg^2+^) and was subsequently incubated at 37°C in a 5% CO_2_ atmosphere for 2 h. To dissolve the formazan crystals, 100 μL of extraction buffer (5% SDS in N,N-Dimethylformamide) was then added to each well, followed by another 2 h of incubation at 37°C with 5% CO_2_. The absorbance of the formazan products in each well was assessed at a wavelength of 570 nm using the Infinite^®^ 200 PRO multi-well plate reader from Tecan Group Ltd. in Männedorf, Switzerland after 2 h of incubation. The relative cell viability index was derived by dividing the absorbance at a given doxorubicin concentration over the absorbance recorded for the control cells (which were incubated without doxorubicin).

### 2.7 Quantitative RT-PCR analysis

To assess the impact of the mechanical treatment on gene expression was evaluated as follows: after the 24-h cyclic stretch application, treated SAOS-2 cells and the static control counterpart were detached using trypsin, pelleted, and prepared for analysis of specific target genes: *SOD1*, *SITR1*, *Nrf2*, and *FOXO1*. RNA was extracted from cellular pellets following the manufacturer’s protocol using the TRIZOL Reagent from Roche Diagnostics GmbH in Mannheim, Germany. The quality of the extracted RNA was evaluated by measuring the absorbance ratio at 260 and 280 nm using the NanoQuant Plate with an Infinite^®^200 PRO multi-well plate reader from Tecan Group Ltd., in Männedorf, Switzerland. The RNA was reverse-transcribed using a SensiFAST™ cDNA Synthesis Kit (Bioline, Meridian Bioscience, London, United Kingdom) in accordance with the manufacturer’s specifications. Gene expression levels were quantified using iTaq Universal SYBR Green Supermix (Biorad Laboratories, Hercules, CA, United States). Quantitative real-time PCR (qRT-PCR) was conducted utilizing a LightCycler 96 Real-Time PCR System (Roche Diagnostics GmbH). For data analysis, the expressions of all target genes were normalized using the ΔΔ cycle threshold method, with human glyceraldehyde 3-phosphate dehydrogenase (GAPDH) gene expression serving as the reference for normalization. The relative quantification was performed by using LightCycler^®^96 system software version 1.1 (Roche). Primers were designed by using the UCSC genome browser (https://genome.ucsc.edu/), ApE software (version 2.0 70.0), NetPrimers software (https://www.premierbiosoft.com/netprimer/), and primer-BLAST (https://www.ncbi.nlm.nih.gov/tools/primer-blast/). The primers were then synthesized by Merck (Life Sciences, Milano, Italy). The sequences of the primers used for quantitative real-time PCR analysis of gene expression in SAOS-2 cells are as in Table 1.

**TABLE 1:** Primer sequences used for RT-PCR analyses.

Gene	Sequences (5′–3′)
*h SOD1 F*	GTG​TGG​CCG​ATG​TGT​CTA​TTG
*h SOD1 R*	TTC​CAG​CGT​TTC​CTG​TCT​TTG
*h SIRT1 F*	TAG​CCT​TGT​CAG​ATA​AGG​AAG​GA
*h SIRT1 R*	ACA​GCT​TCA​CAG​TCA​ACT​TTG​T
*h Nrf-2 F*	TGA​GGT​TTC​TTC​GGC​TAC​GTT
*h Nrf-2 R*	CTT​CTG​TCA​GTT​TGG​CTT​CTG​G
*h GAPDH F*	AGA​AGG​CTG​GGG​CTC​ATT​T
*h GAPDH R*	AGG​GGC​CAT​CCA​CAG​TCT​T

Results are presented as means ± SEM, and variations between the means were assessed using the parametric t-test with the assistance of the GraphPad Prism 9.01 software (San Diego, CA, United States).

## 3 Results

### 3.1 Mechano-induced changes of osteosarcoma SAOS-2 cell metabolome

**FIGURE 1:**
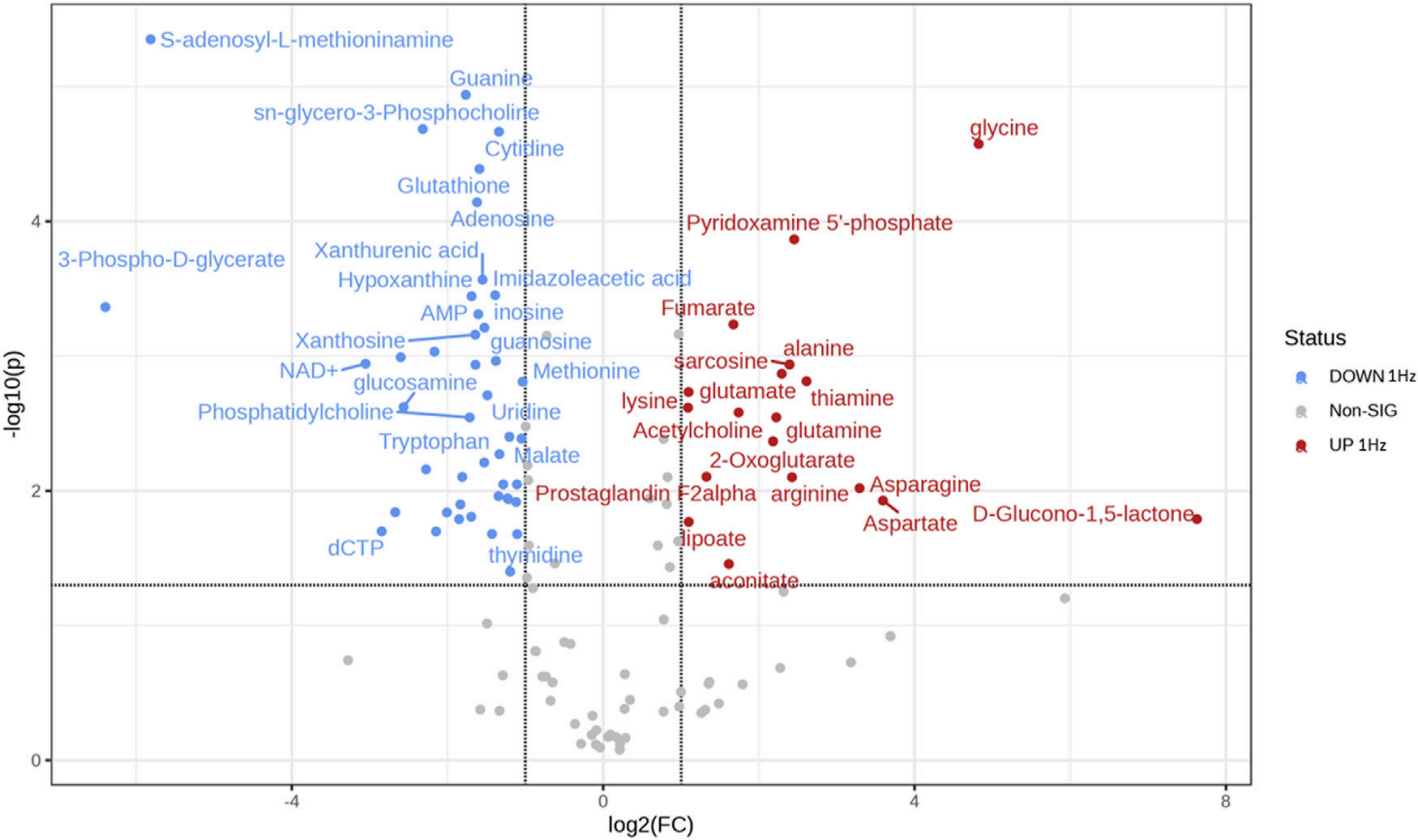
Volcano plot showing the distribution of the fold changes in metabolite concentrations induced by the 24 h 1 Hz cyclic stretch of SAOS-2 cells. Blue and red dots refer to decreased and increased metabolites, respectively, in the 1 Hz samples (fold change >1.5 and adjusted *p*-value FDR < 0.05).

**FIGURE 2:**
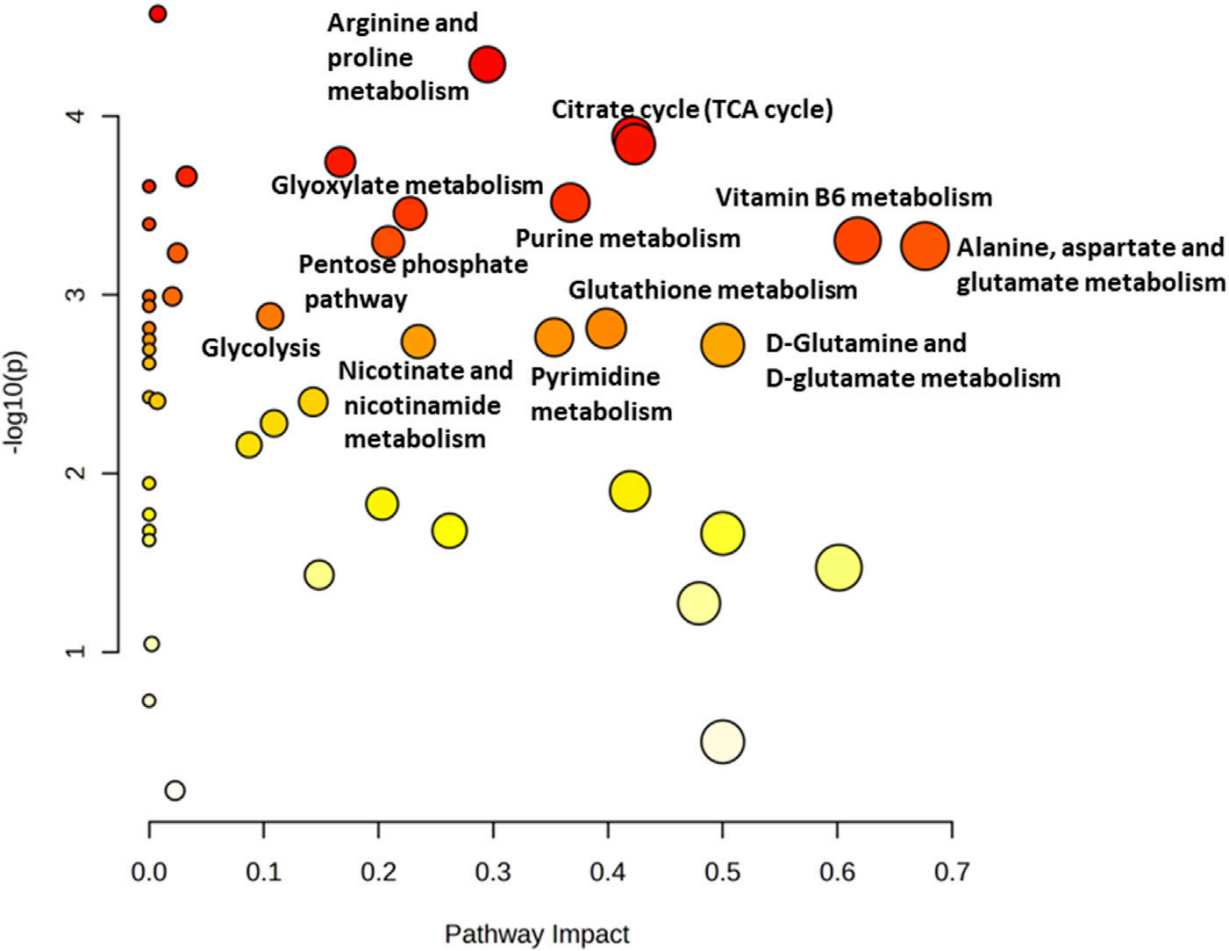
Metabolomics Pathway Analysis (MetPA) of mechanically 1 Hz stimulated SAOS-2 cell line. The color of each circle is based on *p*-values (darker colors indicate more significant changes of metabolites in the corresponding pathway). In contrast, the circle size corresponds to the pathway impact score. The most impacted pathways (high statistical significance scores) are annotated by their full name.

Osteosarcoma SAOS-2 cells were exposed to a specific medium-magnitude strain regimen, which has been considered a valuable insult for understanding important cellular aspects in osteosarcoma cell biology *in vitro* (Alloisio et al., 2023). Specifically, SAOS-2 cells were subjected or not to a 0.5% elongation uniaxial cyclic stretch at 1 Hz frequency for 24 h, and an untargeted metabolomics analysis was performed to evaluate cell metabolism modifications induced by this mechanical stimulation. The significant discriminating metabolites between unstimulated (Ctrl) and mechanically stimulated (1 Hz) SAOS-2 cells were identified using the Volcano plot analysis (Figure 1). Based on the selected criteria, 48 metabolites were significantly downregulated, whereas 25 were significantly upregulated, in the mechanically stretched cells (1 Hz samples) compared to unstimulated SAOS-2 cells (Ctrl samples). Among metabolites that mainly displayed changes in 1 Hz-exposed samples compared to the controls, there were amino acids such as glycine, glutamate, glutamine, aspartate, arginine, and lysine. Their increased level suggests a central role of amino acids in energy production and nucleotide synthesis in cyclically stretched samples (1 Hz) with respect to the untreated specimens (Ctrl). Indeed, the KEGG pathway analysis showed that the significant perturbed metabolic pathways in osteosarcoma SAOS-2 cell lines included: energy metabolism (glycolysis, pentose phosphate pathway, and citrate cycle); alanine, aspartate and glutamate metabolism; purine metabolism; pyrimidine metabolism; and vitamin B6 metabolism (Figure 2).

**FIGURE 3:**
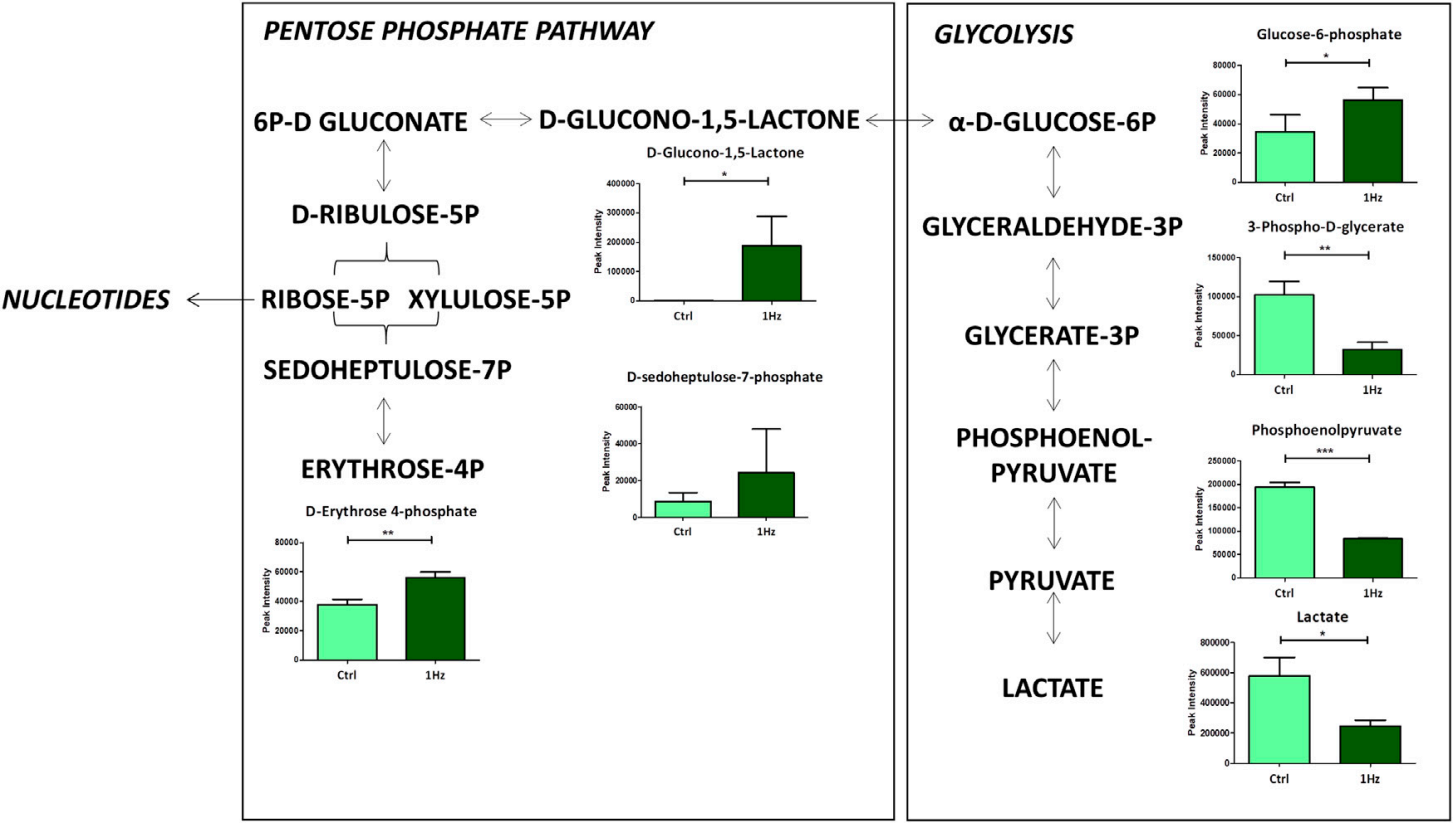
Changes in accumulation of intermediates belonging to glycolysis and Pentose Phosphate Pathway in mechanically stimulated (1 Hz) samples compared to the control (Ctrl). Data are presented as mean ± standard deviation. The statistical significance of each metabolite between samples was determined using Student’s t-test. *p-value < 0.05; **p-value < 0.01; ***p-values < 0.001.

To study in detail the mechanical regulation of the uniaxial cyclic stretch on the energy metabolism, the glycolysis, the Pentose Phosphate Pathways (PPP), and the citrate cycle (TCA) of SAOS-2 cells exposed or not to a 0.5% elongation uniaxial cyclic stretch for 24 h were compared. As Figure 3 clearly shows, we observed a mechanically-induced increased abundance of glucose-6-phosphate (G6P) in mechanically stretched samples (1 Hz). In contrast, lactate accumulation significantly decreased upon the stimulation, confirming that the cyclic stretch application can reverse the Warburg effect in SAOS-2 cells. Since PPP and glycolysis are metabolically linked for sharing the common intermediate G6P, increased glycolysis concurrently leads to decreased PPP rate and *vice versa*. As expected, we highlighted a glucose diversion from the glycolysis into the PPP in 1 Hz stimulated samples.

**FIGURE 4:**
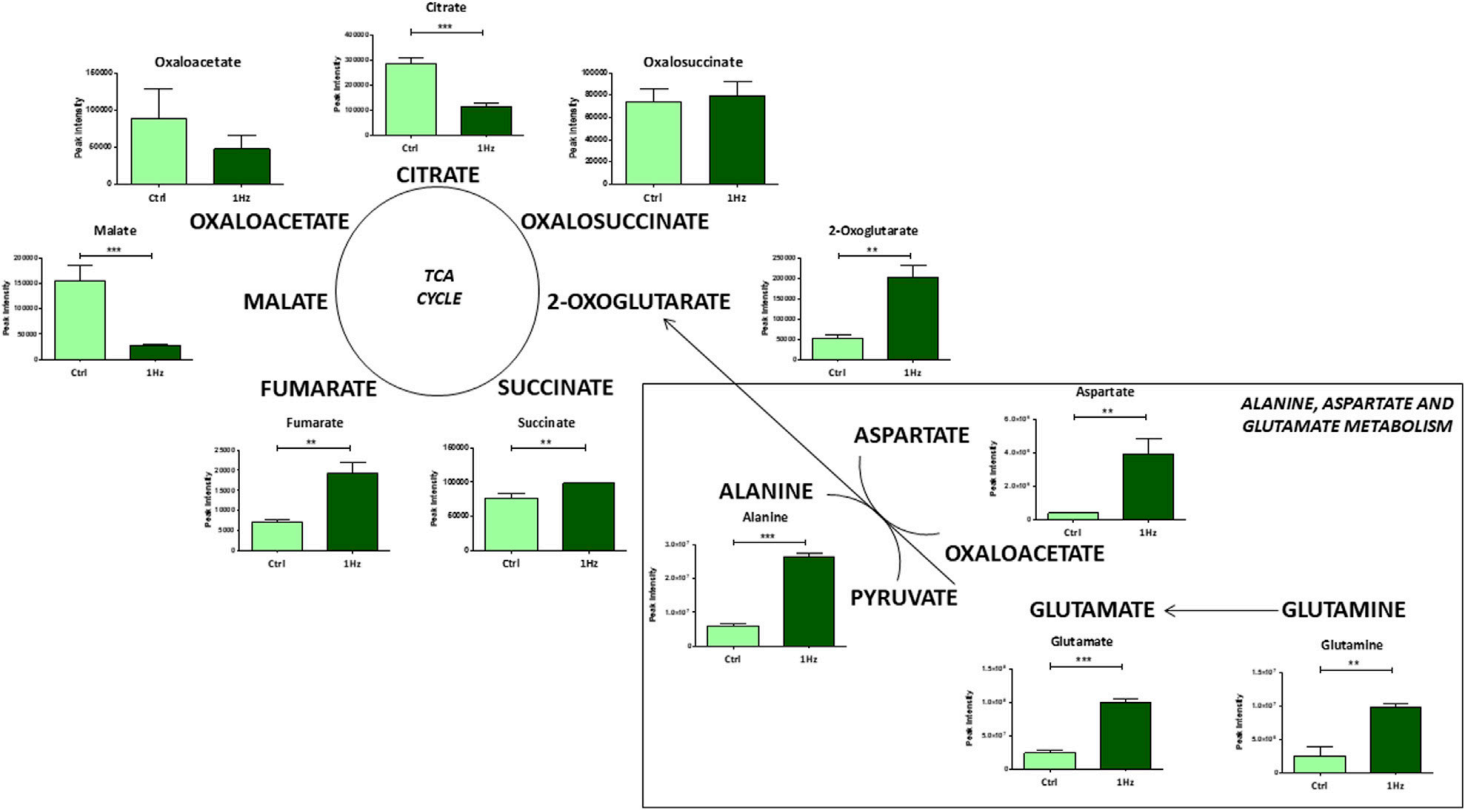
Changes in accumulation of TCA intermediates and glutaminolysis in mechanically stimulated (1 Hz) samples compared to the control (Ctrl). Data are presented as mean ± standard deviation. The statistical significance of each metabolite between samples was determined using Student’s t-test. *p-value < 0.05; **p-value < 0.01; ***p-values < 0.001.

Besides, a significant mechanically-induced decrease in citrate and an increased level of 2-oxoglutarate suggests that in 1 Hz samples TCA metabolites derive from glutaminolysis, the process by which cells convert glutamine into glutamate that can be further metabolized to 2-oxoglutarate to enter the TCA cycle. This last step involves the activity of different transaminases that directly transfer amino groups from glutamate to alpha-keto acids, either oxaloacetate or pyruvate, to generate, respectively, the 2-oxoglutarate/aspartate and 2-oxoglutarate/alanine couples as products (Figure 4).

**FIGURE 5:**
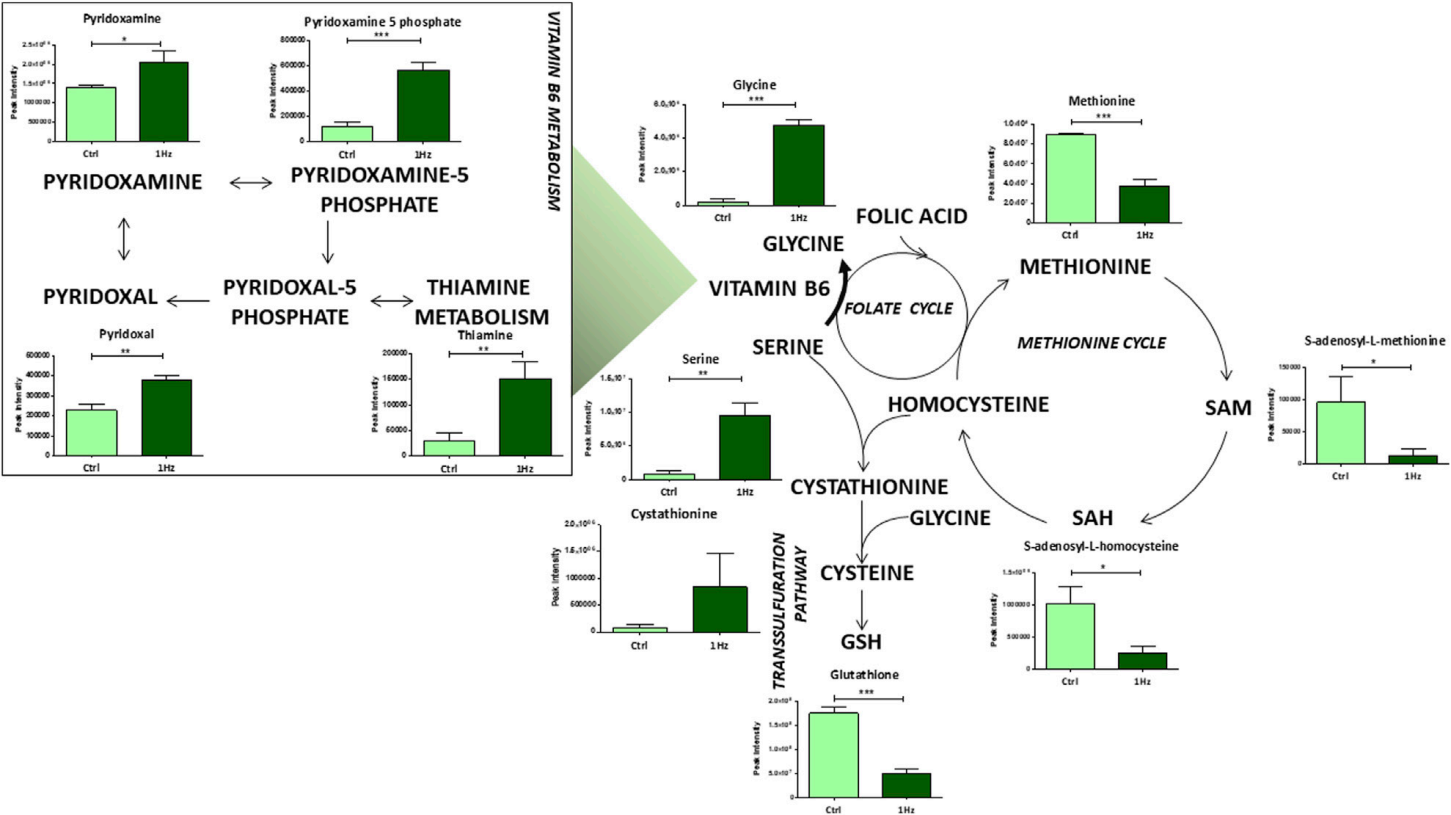
Mechanically-induced changes in the accumulation of intermediates belonging to aminoacid and vitamin B6 metabolisms. Metabolite quantification is reported for the mechanically stimulated SAOS-2 cells (1 Hz) and the static control counterpart (Ctrl). Data are presented as mean ± standard deviation. The statistical significance of each metabolite between samples was determined using Student’s t-test. *p-value < 0.05; **p-value < 0.01; ***p-values < 0.001. The bold arrow indicates the reaction that requires vitamin B6 as a cofactor.

Noteworthy differences were also detected at the level of some individual amino acid concentrations and their relative pathways in the 1 Hz treated SAOS-2 cells compared to the untreated counterpart. For example, glutathione metabolism resulted to be mechanically downregulated. The transsulfuration pathway significantly contributes to glutathione production; it involves the conversion of homocysteine, derived from methionine and folate cycles, into cysteine and reduced glutathione via the intermediate cystathionine. Interestingly, our results showed a downregulation of metabolites implicated in the methionine cycle in 1 Hz samples compared to controls (Figure 5). Besides the above-mentioned amino acids, serine and glycine were also required in these metabolisms. The conversion of serine to glycine involves vitamin B6 as essential coenzyme and generates one-carbon units that enter both the folate and methionine cycle. These pathways are important for nucleotide synthesis and methyl donor S-Adenosyl Methionine (SAM) generation, respectively. Therefore, the observed mechanically-induced upregulation of serine, glycine and bioactive forms of vitamin B6 (pyridoxamine, pyridoxamine 5 phosphate, and pyridoxal) allowed us to suppose that these metabolites do not feed the methionine cycle through the folate cycle. This hypothesis is also corroborated by the reduced levels, in the 1 Hz sample with respect to the control specimen, of SAM and S-adenosyl homocysteine that therefore do not participate in nucleotide synthesis and GSH production.

**FIGURE 6:**
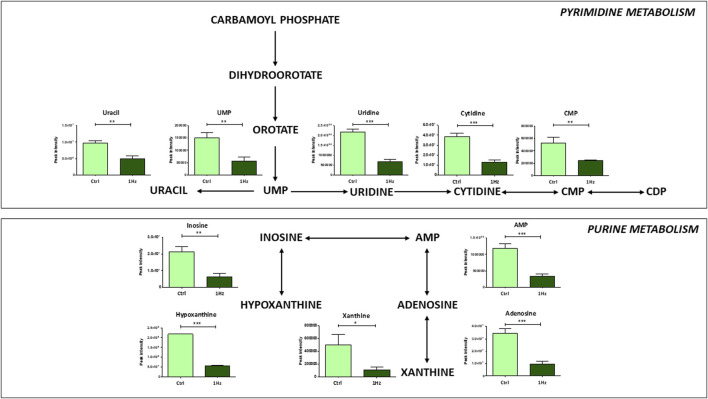
Mechanically-induced changes in the accumulation of intermediates belonging to pyrimidine and purine metabolisms in mechanically stimulated (1 Hz) samples compared to the control (Ctrl). Data are presented as mean ± standard deviation. The statistical significance of each metabolite between samples was determined using Student’s t-test. *p-value < 0.05; **p-value < 0.01; ***p-values < 0.001.

In connection with the alteration of specific amino acid abundance levels, our findings revealed a downregulation, in 1 Hz samples, of purine and pyrimidine metabolisms along with some of their related metabolites (e.g., hypoxanthine, AMP, and adenosine for purine metabolism; UMP, uridine and CMP for pyrimidine metabolism) (Figure 6). Since nucleotide metabolism is essential for the biosynthesis of DNA and RNA, cell signaling, enzyme regulation and proliferation of cancer cells, these inhibitions in 1 Hz samples confirmed that the treatment regulates the *de novo* synthesis process.

### 3.2 1 Hz stretching treatment increased cellular ROS production and SAOS-2 cell sensitivity to doxorubicin

**FIGURE 7:**
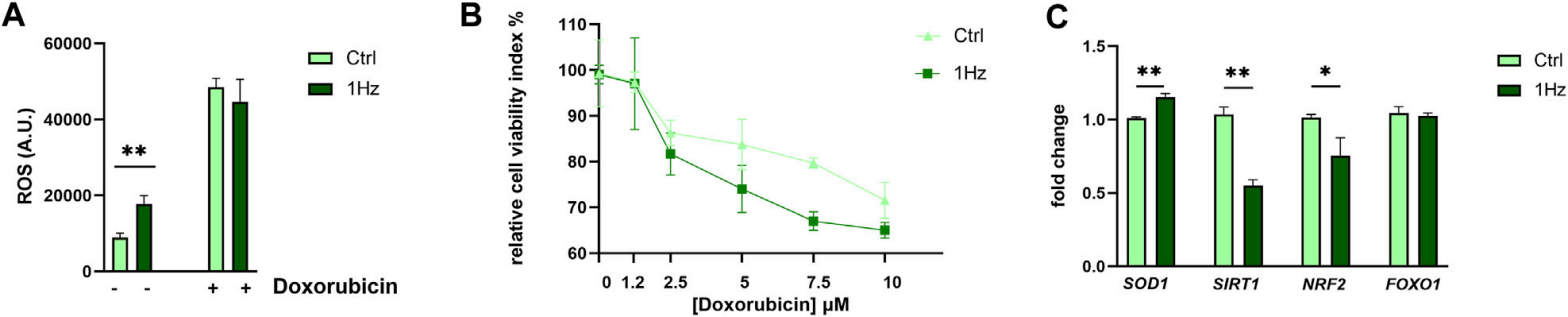
Mechanically-induced upregulation of ROS and Cytotoxicity in SAOS-2 cell line. **(A)** Cellular ROS production for the 1 Hz-stimulated or not stimulated cells is reported both in the presence and the absence of 8,6 µM Doxorubicin treatment. **(B)** Doxorubicin Cytotoxicity for SAOS-2 osteosarcoma cells that were or were not pre-mechanically treated. Light green square symbols represent relative cell viability for cells cyclically stimulated for 24 h at 1 Hz frequency. Dark-green triangles represent control static cells (cells cultured on a silicone support subjected to the same experimental conditions but not stretched). Statistics have been performed on three biological replicates with four technical replicates per condition. **(C)** The impact of a 24 h 1 Hz uniaxial stimulation on the gene expression of four cytoprotective genes (i.e., *SOD1*, *SIRT1*, *Nrf2*, and *FOXO-1*). Statistical analyses were performed on three biological replicates with at least three technical replicates per condition. Statistical significance between treated and control samples was determined using Student’s t-test. *p-value < 0.05; **p-value < 0.01.

Since our metabolomic analysis displayed several distinctive features of increased oxidative stress in mechanically treated SAOS-2 cells (e.g., GSH depletion, pentose phosphate pathway upregulation, and nucleotide synthesis decrease), we evaluated whether the 24 h-1 Hz mechanical stimulation was accompanied or not by an intercellular ROS production. Figure 7A shows that the mechanical stimulation induced a moderate ROS overproduction by 1.89 folds with respect to the static control counterpart. To evaluate a possible synergistic effect of mechanical and chemical stimuli on ROS generation, the mechanically pre-treated cells were incubated with the ROS inducer doxorubicin, a chemotherapeutic drug currently used in osteosarcoma treatment. The concurrent mechanical and chemical stimulations showed a boost in ROS production; Figure 7A displays that the 24 h treatment of SAOS-2- cells with 8.6 µM doxorubicin increases the ROS generation by 5-folds. Therefore, the chemically induced production of ROS masked the differences between static and stretch-induced regulation of ROS production in SAOS-2 cells. Based on the achieved results, we also hypothesized that the stretch-induced disruption of oxidative stress homeostasis could lead to a possible cytotoxic sensitization of the mechanical pre-treated SAOS-2 cells. Therefore, we employed the doxorubicin chemotherapeutic drug to also test the osteosarcoma cell resistance to death. Pre-stretched and control cells were treated with different concentrations of doxorubicin within the 1.25–10 µM range. Figure 7B shows that SAOS-2 cells died in a dose-dependent manner in response to the doxorubicin treatments in both cases. However, the mortality was more effective for the pre-stretched cells. This indicates that 1 Hz-24 h stretch treatment can render osteosarcoma cells more sensitive to doxorubicin-induced death. Since *SOD1*, *SIRT1*, *Nrf2*, and *FOXO-1* are mechanosensitive genes whose proteins have been reported to play an important role in counteracting oxidative stress (Kajihara et al., 2006; Pardo et al., 2008; Chen et al., 2018; Barrera et al., 2021; Chen et al., 2021; Kuno et al., 2023), their gene expression levels were measured using real-time PCR. Figure 7C shows that our 24 h-1 Hz mechanical stimulation did not significantly perturb the expression level of the *FOXO1* gene. On the contrary, the mechanical stimulation induced an upregulation of the *SOD1* gene and a concurrent downregulation of the two transcription factor genes (i.e., *SIRT1* and *Nrf2*) in the mechanically stimulated sample with respect to the control cells.

## 4 Discussion

In recent years, there has been a growing fascination with unraveling the role of mechanical forces in governing cellular biology, with particular emphasis on their impact on cancer cells. Mechanical cues originating from the tumor microenvironment play a pivotal role in shaping cell mechanics and affecting cellular metabolism, ultimately fostering the aggressiveness of cancer. Numerous studies have highlighted that particular metabolic activities can provide critical support for the biological processes within tumors, facilitating both cancer initiation and progression (Hosios et al., 2016; Weber, 2016; Chen et al., 2020; Fritsche-Guenther et al., 2020). Moreover, during cancer progression, there is evidence that mechanical changes from the microenvironment induce crucial molecular signals to guide cells in capturing nutrients to support their metabolic needs (Bays et al., 2017; Bertero et al., 2019; Evers et al., 2021; Torrino et al., 2021). The findings from mechanobiological studies hold the potential to revolutionize current cancer treatment strategies, ultimately leading to improved patient outcomes.

In this study, comparative metabolite characterization by means of untargeted metabolomics analyses was performed to shed light on the molecular mechanisms underlying the mechanical stimulation of the SAOS-2 cell line that could possibly be helpful in clinical therapy for osteosarcoma. We identified several mechanosensitive metabolites that could represent novel oncogenic targets. The intermediates and end-products of glycolysis were found significantly decreased in mechanically stimulated (1 Hz) samples. For rapid growth, cancer cells enhance their metabolism by “metabolic reprogramming” (Chen et al., 2020). Via the well-known “Warburg effect” manifested by increased glucose uptake and lactate production (Hsu and Sabatini, 2008), cancer cells obtain energy either in oxygen or hypoxia (Warburg, 1956), and consequently leads to a downregulation of the TCA cycle (Gatenby and Gillies, 2004). According to many studies, one of the most common metabolic changes in OS is enhanced glycolysis; many glycolytic enzymes have been shown to promote the tumorigenic activity of OS cells and are associated with poor prognosis in patients (Sottnik et al., 2011; Zhuo et al., 2015; Shen et al., 2019; Feng et al., 2022). Metabolic inhibitors of glycolysis (such as 2-deoxy-D-glucose) and of the mitochondrial respiratory pathway (such as metformin) may represent a potentially effective therapy against Ewing Sarcoma cell lines *in vitro* (Dasgupta et al., 2017).

Interestingly, SAOS-2 cells showed an over-accumulation of pentose phosphate pathway (PPP) intermediates after our mechanical stimulation. The regulatory network of PPP flux represents a crucial metabolic adaptation in a number of environmental contexts in human malignancies, including cancer (Jin and Zhou, 2019). It has been reported that in cancer cells, the pentose phosphate pathway (PPP) can concurrently sustain: i) tumor cell survival by providing NADPH, which is needed for fatty acid synthesis under stress conditions, and ii) cell proliferation by the generation of pentose phosphates for DNA synthesis (Patra and Hay, 2014; Jin and Zhou, 2019). In the case of osteosarcoma, Fritsche-Guenther et al. have shown reduced label incorporation of ribose-5-phosphate in both malignant and metastatic *in vitro* cultured OS cells compared to benign cells, suggesting a decreased flux through the PPP (Fritsche-Guenther et al., 2020). Consistent with these findings, our work suggests that mechano-unstimulated osteosarcoma cells do not use PPP for proliferation. Considering that the NADPH generated by PPP also serves to counteract ROS generation, it is reasonable to hypothesize that the increased PPP activity we observed in 1 Hz treated samples may represent a cell response to elevated levels of oxidative stress. Another metabolic aspect to consider is that the conversion of most pyruvate to lactate results in restricting pyruvate entry into the TCA cycle for a higher ATP yield. Consequently, a process known as glutaminolysis (Hosios et al., 2016) alternatively provides the carbon source for the TCA cycle. Several studies have provided substantial evidence regarding the significant role of metabolites derived from glutamine in fueling the tricarboxylic acid (TCA) cycle within cancer cells (Gaglio et al., 2011; Zhao et al., 2019). Consequently, the targeting of glutaminolysis has emerged as a promising strategy for disrupting cancer metabolism and impeding tumor progression (Lukey et al., 2013; Matés et al., 2020; Wang et al., 2020). Along with the mechano-activation of the glutamine catabolism reported for a breast cancer cell model by hyper loading forces (Torrino et al., 2021), our data demonstrate that mechanical stimulation activates glutaminolysis in osteosarcoma cell model suggesting that this typical tumor-associated metabolotype is mechanosensitive.

However, one of the most significant results of our study, which is probably related to this preferential use of glutamine as an anaplerotic substrate for attempting to maintain the TCA cycle flux, was the evident disruption of the oxidative homeostasis in mechano-stimulated SAOS-2 cells when compared to controls. In fact, Gln is a vital precursor of Glu for GSH synthesis and significant decreases in GSH synthesis rates were registered in the mechanically treated cells (1 Hz samples), indicating an exacerbated oxidative stress response which is also sustained by increased levels of *SOD1* gene. Nowadays the high level of oxidative stress is considered a valuable target for anticancer therapy (Benhar et al., 2016; Galadari et al., 2017; Desideri et al., 2019). This effect can be triggered by elevating external reactive oxygen species (ROS) levels and/or by suppressing the intrinsic protective antioxidant system (Van Loenhout et al., 2020). Consistent with previous findings, the current work observed a mechanically-induced increase of ROS generation and a concurrent inhibition of the endogenous protective antioxidant system such as GSH that, in turn, disrupts the cell antioxidant defenses. Numerous reports have shown that increased oxidative stress can inhibit cancer progression and metastasis by activating different cell death pathways and that the GSH may promote tumorigenesis and resistance to therapy (Benhar et al., 2016; Galadari et al., 2017). As previously reported, the depletion of the GSH antioxidant system can also be attributed to lower fluxes through the cysteine route (Desideri et al., 2019). Compared to controls, decreased levels of metabolites implicated in methionine and folate cycles were observed in mechanically treated samples. The folate and methionine pathways regulate the amino acid synthesis, purine, and pyrimidine metabolism, S-adenosyl-homocysteine levels and methylation capacity, as well as the maintenance of redox homeostasis through GSH, ATP, and NADH generation (Shuvalov et al., 2017) so they were considered key players in the evolution of cancer (Ducker and Rabinowitz, 2017) and can be an effective target for cancer treatment (Rosenzweig et al., 2018; Sullivan et al., 2021). Since the one-carbon metabolism, which is generally believed to sustain tumor cell survival and growth (Newman and Maddocks, 2017), was downregulated in our 1 Hz samples, our mechanical regimen of stimulation may be considered as a possible oncosuppressor treatment.

It is well known that, along with glutamine and cysteine, other amino acids, such as glycine and serine, are utilized to synthesize GSH and therefore required to maintain the cellular redox balance (Amelio et al., 2014). Glycine production depends on serine availability and on the vitamin B6 cofactor. All these three metabolites were upregulated in mechanically treated samples. Elevated circulating amounts of antioxidant B6 vitamers were correlated with a reduced incidence of several distinct neoplasms (Galluzzi et al., 2013). Moreover, vitamin B6 is reported to sensitize cancer cells to apoptosis induction by distinct types of physical and chemical stress, including several chemotherapeutics (Galluzzi et al., 2012). The antioxidant properties of vitamin B6 can derive from its direct involvement in reactions with ROS (Ohta and Foote, 2002; Matxain et al., 2006) and are indeed linked to its role as an enzyme cofactor in the transsulfuration pathway. In fact, it is known that vitamin B6 and folate deficiency can lead to elevated homocysteine levels, which in turn generate ROS (Tinelli et al., 2019). On the other hand, our results draw attention to the fact that the vitamin B6-dependent accumulation of glycine and serine in mechanically stimulated SAOS cells does not supply the methionine cycle through the folate cycle, leading to a reduction of purine and pyrimidine production. Cancer cells must utilize large amounts of energy and nucleotides for DNA and RNA synthesis, consequently, an upregulation of the *de novo* nucleotide metabolism enables cells to proliferate rapidly (Wu et al., 2022) Several intermediate metabolites of the purine nucleotide *de novo* synthesis pathway were found to be significantly increased in OS patients, indicating that the active purine metabolism was closely related to the development of osteosarcoma (Lv et al., 2020). These observations are in line with our metabolomic analysis where purine and pyrimidine metabolites were detected at lower concentrations in mechanically treated samples when compared to controls, which might possibly underlie the cytotoxic sensitization induced by the mechanical pre-treatment.

Furthermore, several studies have shown that elevated cytoprotective gene expression is a necessary survival adaptation during tumor progression. It is widely accepted that chemotherapeutics’ anticancer effectiveness enables cells to cope with increased cellular and extracellular redox stress (Kajihara et al., 2006; Lee et al., 2013; Barrera et al., 2021; Kuno et al., 2023). Specifically, *SIRT1* is reported to play a distinctive role in osteosarcoma tumors, promoting metastasis, with its inhibition exerting antitumor activity. In human osteosarcoma cells, *SIRT1* expression level may be coupled with metastatic risk in patients with osteosarcoma (Cheng et al., 2013; Zhang et al., 2016; Singh et al., 2018; Fathizadeh et al., 2019). Interestingly, *Nrf2* activation is associated with poor prognosis, and Nrf2 has been identified as a key activator of cancer-supportive anabolic metabolism (He et al., 2020). Emerging evidence from several groups now indicates that *SOD1* is overexpressed in cancers, and the activity of SOD1 may be essential to maintaining cellular ROS below a critical threshold (Papa et al., 2014).

The present study reveals that the expression levels of *SOD1*, *SIRT1*, and *Nrf2* genes, which are reported to be cytoprotective (Kajihara et al., 2006; Pardo et al., 2008; Lee et al., 2013; Chen et al., 2018; Barrera et al., 2021; Chen et al., 2021; Kuno et al., 2023), can be mechanically modulated. Notably, the downregulation of osteosarcoma-supporting genes *SIRT1* and *Nrf2*, as demonstrated in the mechanically treated specimens in the current study, aligns with the compromised protection of SAOS-2 cells induced by mechanical stimulation. The intertwining between mechanosensing and cell cytotoxic sensitization envisages the possibility that the mechanosensitive molecules could be targeted for new therapeutic strategies in osteosarcoma. In this regard, our data report that the mechano-disruption of oxidative stress homeostasis correlated with a cytotoxic sensitization (to the doxorubicin-induced cell death) of the mechanical pre-treated SAOS-2 cells.

In conclusion, current LC-MS-based metabolomics profiling revealed different significantly altered metabolites in SAOS-2 cells after mechanical 1 Hz stimulation. Pathway analysis suggested a disrupted energy metabolism in mechanically treated cells, characterized by a significant downregulation of both glycolysis and TCA cycle, but also a dysregulation of amino acid metabolism (i.e., enhanced glutaminolysis, reduced methionine cycle). As supported by the mechanically-induced GSH depletion and ROS enrichment, in turn, our data show that our mechanical regiment can induce an increase in the levels of oxidative stress homeostasis which sensitizes SAOS-2 cells to doxorubicin-induced cell death.

**FIGURE 8:**
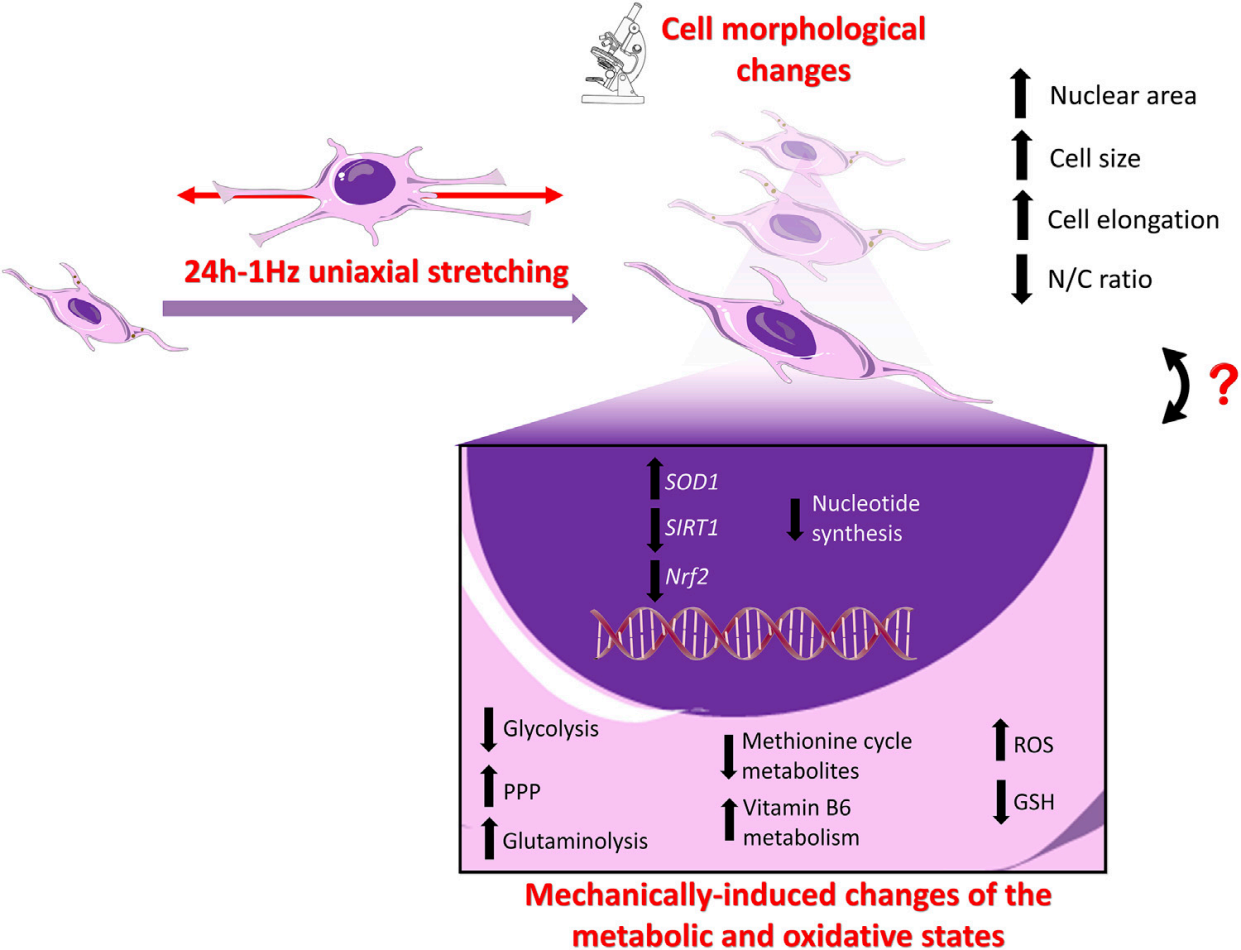
Schematic Illustration of the stretch-induced changes in SAOS-2 cells: **Upper panel**. Cell Morphology: Our previous findings using confocal and atomic force microscopy (AFM) revealed that the application of cyclic stretch induces significant alterations in the morphology of SAOS-2 cells (Alloisio et al., 2023). Notably, the nucleus area, recognized for its crucial role in mechano-regulating cell behaviors, undergoes enlargement. The entire cell undergoes increased elongation post-cyclic stretch application, resulting in a noteworthy increase in size and disruption of the nuclear-to-cellular (N/C) ratio of SAOS-2 cells (Alloisio et al., 2023). **Lower panel**. Gene Expression Levels: While the expression levels of osteogenic differentiative markers (i.e., *ALP*, *COL1*, *RUNX-2*) remain unaffected by mechanical stimulation (Alloisio et al., 2023), the present data shows that *SIRT1* and *Nrf2* are downregulated, whereas *SOD1* is upregulated in the mechanically treated samples compared to the untreated specimens. Metabolic State: Mechanical stimulation is associated with a reversal of the Warburg effect, upregulation of glutaminolysis, GSH depletion, intracellular ROS accumulation, and upregulation of PPP, coupled with downregulation of nucleotide synthesis.

As a whole, our research group has demonstrated that subjecting SAOS-2 cells to 1Hz-24 h stretch results in significant morphological, metabolic, and functional alterations. These changes exhibit a dual impact, fostering a pro-metastatic state characterized by heightened cell migration (Alloisio et al., 2023) while simultaneously downregulating defence mechanisms against doxorubicin-induced death. Figure 8 attempts to summarize the mechanically-induced changes of SAOS-2 cells, providing a schematic representation of the potential interconnections between the metabolic state and gene expression, intricately linked to concurrent variations in cell morphology (Alloisio et al., 2023). The coupling of cell structure with metabolism and function poses a challenge, with spatiotemporal cell deformations influencing cellular responses and malignancy (Muff et al., 2007; Cascione et al., 2019; Luo et al., 2022; Tollis et al., 2022). While it has been proposed that the structural components of the cell utilize cellular energy to orchestrate cellular functions, acting as a dynamic bridge between thermodynamics and gene expression (Pienta and Hoover, 1994; Bays et al., 2017; Bertero et al., 2019; Evers et al., 2021; Torrino et al., 2021), a comprehensive understanding of the intricate interplay between cell structure, cellular energy, and function remains imperative to properly connect changes in the metabolic state with cell morphological alterations. Nuclear morphology changes, often documented in cancer cells, may impact chromatin organization and gene expression, particularly in tumor development and cancer progression (Jevtić et al., 2014). In the context of mechanical-induced changes observed in SAOS-2 cells, it is conceivable that the enlargement of nuclear size may be associated with chromatin reorganization and gene expression. However, this hypothesis necessitates thorough validation in subsequent studies.

These findings contribute to a nuanced understanding of the intricate responses of SAOS-2 cells to mechanical stimuli, providing valuable insights into potential implications for cell behavior, metastasis, and sensitivity to therapeutic agents. Nevertheless, the authors acknowledge the limitations of their study focusing on a single cell line and recognize the importance of further investigations using multiple cell lines to expand the scope of research in cancer mechanobiology. A comprehensive understanding of mechanobiology and its impact on cell metabolism in various cancer types can provide valuable insights for the development of more effective and targeted approaches in cancer treatment. By identifying commonalities and differences in the response of different cancer cell lines to mechanical forces, researchers can potentially uncover new therapeutic targets and strategies for combating cancer.

## Data Availability

The data presented in the study are deposited in the MetaboLights repository, accession number MTBLS9560.

## References

[B1] Adamopoulos C. Gargalionis A. N. Piperi C. Papavassiliou A. G. (2017). Recent advances in mechanobiology of osteosarcoma. J. Cell. Biochem 118, 232–236. 10.1002/jcb.25660 27463370

[B2] Alloisio G. Ciaccio C. Fasciglione G. F. Tarantino U. Marini S. Coletta M. (2021). Effects of extracellular osteoanabolic agents on the endogenous response of osteoblastic cells. Cells 10, 2383. 10.3390/cells10092383 34572032 PMC8471159

[B3] Alloisio G. Rodriguez D. B. Luce M. Ciaccio C. Marini S. Cricenti A. (2023). Cyclic stretch-induced mechanical stress applied at 1 Hz frequency can alter the metastatic potential properties of SAOS-2 osteosarcoma cells. Int. J. Mol. Sci. 24, 7686. do 10.3390/ijms24097686 37175397 PMC10178551

[B4] Amelio I. Cutruzzolá F. Antonov A. Agostini M. Melino G. (2014). Serine and glycine metabolism in cancer. Trends biochem. Sci. 39, 191–198. 10.1016/j.tibs.2014.02.004 24657017 PMC3989988

[B5] Barrera G. Cucci M. A. Grattarola M. Dianzani C. Muzio G. Pizzimenti S. (2021). Control of oxidative stress in cancer chemoresistance: spotlight on Nrf2 role. Antioxidants (Basel) 10, 510. 10.3390/antiox10040510 33805928 PMC8064392

[B6] Bays J. L. Campbell H. K. Heidema C. Sebbagh M. DeMali K. A. (2017). Linking E-cadherin mechanotransduction to cell metabolism through force-mediated activation of AMPK. Nat. Cell. Biol. 19, 724–731. 10.1038/ncb3537 28553939 PMC5494977

[B7] Benhar M. Shytaj I. L. Stamler J. S. Savarino A. (2016). Dual targeting of the thioredoxin and glutathione systems in cancer and HIV. J. Clin. Investig. 126, 1630–1639. 10.1172/JCI85339 27135880 PMC4855928

[B8] Bertero T. Oldham W. M. Grasset E. M. Bourget I. Boulter E. Pisano S. (2019). Tumor-stroma mechanics coordinate amino acid availability to sustain tumor growth and malignancy. Cell. Metab. 29, 124–140. 10.1016/j.cmet.2018.09.012 30293773 PMC6432652

[B9] Cascione M. De Matteis V. Mandriota G. Leporatti S. Rinaldi R. (2019). Acute cytotoxic effects on morphology and mechanical behavior in MCF-7 induced by TiO2NPs exposure. Int. J. Mol. Sci. 20 (14), 3594. 10.3390/ijms20143594 31340471 PMC6678441

[B10] Chen X. Chen S. Yu D. (2020). Metabolic reprogramming of chemoresistant cancer cells and the potential significance of metabolic regulation in the reversal of cancer chemoresistance. Metabolites 10, 289. 10.3390/metabo10070289 32708822 PMC7408410

[B11] Chen X. Yan J. He F. Zhong D. Yang H. Pei M. (2018). Mechanical stretch induces antioxidant responses and osteogenic differentiation in human mesenchymal stem cells through activation of the AMPK-SIRT1 signaling pathway. Free Radic. Biol. Med. 126, 187–201. 10.1016/j.freeradbiomed.2018.08.001 30096433 PMC6165675

[B12] Chen X. Zhu X. Wei A. Chen F. Gao Q. Lu K. (2021). Nrf2 epigenetic derepression induced by running exercise protects against osteoporosis. Bone Res. 9, 15. 10.1038/s41413-020-00128-8 33637693 PMC7910611

[B13] Cheng Y. Cai L. Jiang P. Wang J. Gao C. Feng H. (2013). SIRT1 inhibition by melatonin exerts antitumor activity in human osteosarcoma cells. Eur. J. Pharmacol. 715 (1-3), 219–229. 10.1016/j.ejphar.2013.05.017 23726949

[B14] Dasgupta A. Trucco M. Rainusso N. Bernardi R. J. Shuck R. Kurenbekova L. (2017). Metabolic modulation of Ewing sarcoma cells inhibits tumor growth and stem cell properties. Oncotarget 8, 77292–77308. 10.18632/oncotarget.20467 29100387 PMC5652780

[B15] Desideri E. Ciccarone F. Ciriolo M. R. (2019). Targeting glutathione metabolism: partner in crime in anticancer therapy. Nutrients 11, 1926. 10.3390/nu11081926 31426306 PMC6724225

[B16] Djordjevic D. Jakovljevic V. Cubrilo D. Zlatkovic M. Zivkovic V. Djuric D. (2010). Coordination between nitric oxide and superoxide anion radical during progressive exercise in elite soccer players. Open biochem. J. 4, 100–106. 10.2174/1874091X01004010100 21633721 PMC3104555

[B17] Ducker G. S. Rabinowitz J. D. (2017). One-carbon metabolism in health and disease. Cell. Metab. 25, 27–42. 10.1016/j.cmet.2016.08.009 27641100 PMC5353360

[B18] Dufrêne Y. F. Ando T. Garcia R. Alsteens D. Martinez-Martin D. Engel A. (2017). Imaging modes of atomic force microscopy for application in molecular and cell biology. Nat. Nanotech 12, 295–307. 10.1038/nnano.2017.45 28383040

[B19] Eleutherio E. C. A. Silva Magalhães R. S. de Araújo Brasil A. Monteiro Neto J. R. de Holanda Paranhos L. (2021). SOD1, more than just an antioxidant. Arch. Biochem. Biophys. 697, 108701. 10.1016/j.abb.2020.108701 33259795

[B20] Evers T. M. J. Holt L. J. Alberti S. Mashaghi A. (2021). Reciprocal regulation of cellular mechanics and metabolism. Nat. Metab. 3, 456–468. 10.1038/s42255-021-00384-w 33875882 PMC8863344

[B21] Fathizadeh H. Mirzaei H. Asemi Z. (2019). Melatonin: an anti-tumor agent for osteosarcoma. Cancer Cell. Int. 19, 319. 10.1186/s12935-019-1044-2 31798348 PMC6884844

[B22] Feng Z. Ou Y. Hao L. (2022). The roles of glycolysis in osteosarcoma. Front. Pharmacol. 13, 950886. 10.3389/fphar.2022.950886 36059961 PMC9428632

[B23] Fritsche-Guenther R. Gloaguen Y. Kirchner M. Mertins P. Tunn P.-U. Kirwan J. A. (2020). Progression-dependent altered metabolism in osteosarcoma resulting in different nutrient source dependencies. Cancers (Basel) 12, 1371. 10.3390/cancers12061371 32471029 PMC7352851

[B24] Gaglio D. Metallo C. M. Gameiro P. A. Hiller K. Danna L. S. Balestrieri C. (2011). Oncogenic K‐Ras decouples glucose and glutamine metabolism to support cancer cell growth. Mol. Syst. Biol. 7, 523. 10.1038/msb.2011.56 21847114 PMC3202795

[B25] Galadari S. Rahman A. Pallichankandy S. Thayyullathil F. (2017). Reactive oxygen species and cancer paradox: to promote or to suppress? Free Radic. Biol. Med. 104, 144–164. 10.1016/j.freeradbiomed.2017.01.004 28088622

[B26] Galluzzi L. Vacchelli E. Michels J. Garcia P. Kepp O. Senovilla L. (2013). Effects of vitamin B6 metabolism on oncogenesis, tumor progression and therapeutic responses. Oncogene 32, 4995–5004. 10.1038/onc.2012.623 23334322

[B27] Galluzzi L. Vitale I. Senovilla L. Olaussen K. A. Pinna G. Eisenberg T. (2012). Prognostic impact of vitamin B6 metabolism in lung cancer. Cell. Rep. 2, 257–269. 10.1016/j.celrep.2012.06.017 22854025

[B28] Gatenby R. A. Gillies R. J. (2004). Why do cancers have high aerobic glycolysis? Nat. Rev. Cancer 4, 891–899. 10.1038/nrc1478 15516961

[B29] Gioia M. Michaletti A. Scimeca M. Marini M. Tarantino U. Zolla L. (2018). Simulated microgravity induces a cellular regression of the mature phenotype in human primary osteoblasts. Cell. Death Discov. 4, 59. 10.1038/s41420-018-0055-4 29760957 PMC5945613

[B30] Harris M. A. Hawkins C. J. (2022). Recent and ongoing research into metastatic osteosarcoma treatments. Int. J. Mol. Sci. 23, 3817. 10.3390/ijms23073817 35409176 PMC8998815

[B31] Hattinger C. M. Patrizio M. P. Fantoni L. Casotti C. Riganti C. Serra M. (2021). Drug resistance in osteosarcoma: emerging biomarkers, therapeutic targets and treatment strategies. Cancers (Basel) 13, 2878. 10.3390/cancers13122878 34207685 PMC8228414

[B32] He F. Antonucci L. Karin M. (2020). NRF2 as a regulator of cell metabolism and inflammation in cancer. Carcinogenesis 41 (4), 405–416. 10.1093/carcin/bgaa039 32347301 PMC7298623

[B33] Holle A. W. Engler A. J. (2011). More than a feeling: discovering, understanding, and influencing mechanosensing pathways. Curr. Opin. Biotechnol. 22, 648–654. 10.1016/j.copbio.2011.04.007 21536426 PMC3150613

[B34] Hosios A. M. Hecht V. C. Danai L. V. Johnson M. O. Rathmell J. C. Steinhauser M. L. (2016). Amino acids rather than glucose account for the majority of cell mass in proliferating mammalian cells. Dev. Cell. 36, 540–549. 10.1016/j.devcel.2016.02.012 26954548 PMC4766004

[B35] Hsu P. P. Sabatini D. M. (2008). Cancer cell metabolism: Warburg and beyond. Cell. 134, 703–707. 10.1016/j.cell.2008.08.021 18775299

[B36] Jevtić P. Edens L. J. Vuković L. D. Levy D. L. (2014). Sizing and shaping the nucleus: mechanisms and significance. Curr. Opin. Cell. Biol. 28, 16–27. 10.1016/j.ceb.2014.01.003 24503411 PMC4061251

[B37] Jin L. Zhou Y. (2019). Crucial role of the pentose phosphate pathway in malignant tumors. Oncol. Lett. 17, 4213–4221. 10.3892/ol.2019.10112 30944616 PMC6444344

[B38] Kajihara T. Jones M. Fusi L. Takano M. Feroze-Zaidi F. Pirianov G. (2006). Differential expression of FOXO1 and FOXO3a confers resistance to oxidative cell death upon endometrial decidualization. Mol. Endocrinol. 20, 2444–2455. 10.1210/me.2006-0118 16709600

[B39] Kuno A. Hosoda R. Tsukamoto M. Sato T. Sakuragi H. Ajima N. (2023). SIRT1 in the cardiomyocyte counteracts doxorubicin-induced cardiotoxicity via regulating histone H2AX. Cardiovasc. Res. 118, 3360–3373. 10.1093/cvr/cvac026 35258628

[B40] Lamego I. Duarte I. F. Marques M. P. M. Gil A. M. (2014). Metabolic markers of MG-63 osteosarcoma cell line response to doxorubicin and methotrexate treatment: comparison to cisplatin. J. Proteome Res. 13, 6033–6045. 10.1021/pr500907d 25382592

[B41] Lee K. Briehl M. M. Mazar A. P. Batinic-Haberle I. Reboucas J. S. Glinsmann-Gibson B. (2013). The copper chelator ATN-224 induces peroxynitrite-dependent cell death in hematological malignancies. Free Radic. Biol. Med. 60, 157–167. 10.1016/j.freeradbiomed.2013.02.003 23416365 PMC3654089

[B42] Li W. Zhang H. Assaraf Y. G. Zhao K. Xu X. Xie J. (2016). Overcoming ABC transporter-mediated multidrug resistance: molecular mechanisms and novel therapeutic drug strategies. Drug resist. Updat 27, 14–29. 10.1016/j.drup.2016.05.001 27449595

[B43] Lilienthal I. Herold N. (2020). Targeting molecular mechanisms underlying treatment efficacy and resistance in osteosarcoma: a review of current and future strategies. Int. J. Mol. Sci. 21, E6885. 10.3390/ijms21186885 PMC7555161 32961800

[B44] Lukey M. J. Wilson K. F. Cerione R. A. (2013). Therapeutic strategies impacting cancer cell glutamine metabolism. Future Med. Chem. 5, 1685–1700. 10.4155/fmc.13.130 24047273 PMC4154374

[B45] Luo M. Cai G. Ho K. K. Y. Wen K. Tong Z. Deng L. (2022). Compression enhances invasive phenotype and matrix degradation of breast Cancer cells via Piezo1 activation. BMC Mol. Cell. Biol. 23 (1), 1. 10.1186/s12860-021-00401-6 34979904 PMC8722159

[B46] Lv D. Zou Y. Zeng Z. Yao H. Ding S. Bian Y. (2020). Comprehensive metabolomic profiling of osteosarcoma based on UHPLC-HRMS. Metabolomics 16, 120. 10.1007/s11306-020-01745-4 33210231 PMC7674324

[B47] Maiese K. Chong Z. Z. Shang Y. C. Hou J. (2009). A “FOXO” in sight: targeting Foxo proteins from conception to cancer. Med. Res. Rev. 29, 395–418. 10.1002/med.20139 18985696 PMC2666780

[B48] Matés J. M. Di Paola F. J. Campos-Sandoval J. A. Mazurek S. Márquez J. (2020). Therapeutic targeting of glutaminolysis as an essential strategy to combat cancer. Semin. Cell. Dev. Biol. 98, 34–43. 10.1016/j.semcdb.2019.05.012 31100352

[B49] Matxain J. M. Ristilä M. Strid Å. Eriksson L. A., 2006. Theoretical study of the antioxidant properties of pyridoxine. J. Phys. Chem. A 110, 13068–13072. do 10.1021/jp065115p 17134167

[B50] Michaletti A. Gioia M. Tarantino U. Zolla L. (2017). Effects of microgravity on osteoblast mitochondria: a proteomic and metabolomics profile. Sci. Rep. 7, 15376. 10.1038/s41598-017-15612-1 29133864 PMC5684136

[B51] Mohammed D. Versaevel M. Bruyère C. Alaimo L. Luciano M. Vercruysse E. (2019). Innovative tools for mechanobiology: unraveling outside-in and inside-out mechanotransduction. Front. Bioeng. Biotechnol. 7, 162. 10.3389/fbioe.2019.00162[ 31380357 PMC6646473

[B52] Muff R. Nigg N. Gruber P. Walters D. Born W. Fuchs B. (2007). Altered morphology, nuclear stability and adhesion of highly metastatic derivatives of osteoblast-like SAOS-2 osteosarcoma cells. Anticancer Res. 27 (6B), 3973–3979. PMID: 18225558. 18225558

[B53] Müller D. A. Silvan U. (2019). On the biomechanical properties of osteosarcoma cells and their environment. Int. J. Dev. Biol. 63, 1–8. 10.1387/ijdb.190019us 30919911

[B54] Newman A. C. Maddocks O. D. K. (2017). One-carbon metabolism in cancer. Br. J. Cancer 116, 1499–1504. 10.1038/bjc.2017.118 28472819 PMC5518849

[B55] Ohta B. K. Foote C. S. (2002). Characterization of endoperoxide and hydroperoxide intermediates in the reaction of pyridoxine with singlet oxygen. J. Am. Chem. Soc. 124, 12064–12065. 10.1021/ja0205481 12371824

[B56] Palomero J. Pye D. Kabayo T. Jackson M. J. (2012). Effect of passive stretch on intracellular nitric oxide and superoxide activities in single skeletal muscle fibres: influence of ageing. Free Radic. Res. 46, 30–40. 10.3109/10715762.2011.637203 22103935

[B57] Papa L. Manfredi G. Germain D. (2014). SOD1, an unexpected novel target for cancer therapy. Genes. & cancer 5 (1-2), 15–21. 10.18632/genesandcancer.4 24955214 PMC4063254

[B58] Pardo P. S. Lopez M. A. Boriek A. M., 2008. FOXO transcription factors are mechanosensitive and their regulation is altered with aging in the respiratory pump. Am. J. Physiol. Cell. Physiol. 294, C1056–C1066. do 10.1152/ajpcell.00270.2007 18272820

[B59] Park J. S. Burckhardt C. J. Lazcano R. Solis L. M. Isogai T. Li L. (2020). Mechanical regulation of glycolysis via cytoskeleton architecture. Nature 578, 621–626. 10.1038/s41586-020-1998-1 32051585 PMC7210009

[B60] Park J.-Y. Kim Y. W. Park Y.-K. (2012). Nrf2 expression is associated with poor outcome in osteosarcoma. Pathology 44, 617–621. 10.1097/PAT.0b013e328359d54b 23172081

[B61] Patra K. C. Hay N. (2014). The pentose phosphate pathway and cancer. Trends biochem. Sci. 39, 347–354. 10.1016/j.tibs.2014.06.005 25037503 PMC4329227

[B62] Pienta K. J. Hoover C. N. (1994). Coupling of cell structure to cell metabolism and function. J. Cell. Biochem. 55 (1), 16–21. 10.1002/jcb.240550104 8083296

[B63] Polesskaya A. Vicente-Manzanares M. (2020). Meeting Report - workshop “Actin-based mechanosensation and force generation in health and disease.”. J. Cell. Sci. 133, jcs244. 10.1242/jcs.244319 32184275

[B64] Ren L. Hong E. S. Mendoza A. Issaq S. Tran Hoang C. Lizardo M. 2017. Metabolomics uncovers a link between inositol metabolism and osteosarcoma metastasis. Oncotarget 8, 38541–38553. d 10.18632/oncotarget.15872 28404949 PMC5503552

[B65] Rosenzweig A. Blenis J. Gomes A. P. (2018). Beyond the Warburg effect: how do cancer cells regulate one-carbon metabolism? Front. Cell. Dev. Biol. 6, 90. 10.3389/fcell.2018.00090 30159313 PMC6103474

[B66] Schiliro C. Firestein B. L. (2021). Mechanisms of metabolic reprogramming in cancer cells supporting enhanced growth and proliferation. Cells 10, 1056. 10.3390/cells10051056 33946927 PMC8146072

[B67] Shen Y. Zhao S. Wang S. Pan X. Zhang Y. Xu J. (2019). S1P/S1PR3 axis promotes aerobic glycolysis by YAP/c-MYC/PGAM1 axis in osteosarcoma. EBioMedicine 40, 210–223. 10.1016/j.ebiom.2018.12.038 30587459 PMC6412077

[B68] Shoaib Z. Fan T. M. Irudayaraj J. M. K., 2022. Osteosarcoma mechanobiology and therapeutic targets. Br. J. Pharmacol. 179, 201–217. do 10.1111/bph.15713 34679192 PMC9305477

[B69] Shuvalov O. Petukhov A. Daks A. Fedorova O. Vasileva E. Barlev N. A. (2017). One-carbon metabolism and nucleotide biosynthesis as attractive targets for anticancer therapy. Oncotarget 8, 23955–23977. 10.18632/oncotarget.15053 28177894 PMC5410357

[B70] Singh C. K. Chhabra G. Ndiaye M. A. Garcia-Peterson L. M. Mack N. J. Ahmad N. (2018). The role of sirtuins in antioxidant and redox signaling. Antioxidants redox Signal. 28 (8), 643–661. 10.1089/ars.2017.7290 PMC5824489 28891317

[B71] Sottnik J. L. Lori J. C. Rose B. J. Thamm D. H., 2011. Glycolysis inhibition by 2-deoxy-D-glucose reverts the metastatic phenotype *in vitro* and *in vivo* . Clin. Exp. Metastasis 28, 865–875. do 10.1007/s10585-011-9417-5 21842413

[B72] Sullivan M. R. Darnell A. M. Reilly M. F. Kunchok T. Joesch-Cohen L. Rosenberg D. (2021). Methionine synthase is essential for cancer cell proliferation in physiological folate environments. Nat. Metab. 3, 1500–1511. 10.1038/s42255-021-00486-5 34799701 PMC8608285

[B73] Talayero V. C. Vicente-Manzanares M. (2023). A primer on cancer-associated fibroblast mechanics and immunosuppressive ability. Explor. Target. Antitumor Ther. 4, 17–27. 10.37349/etat.2023.00120 36937319 PMC10017186

[B74] Tinelli C. Di Pino A. Ficulle E. Marcelli S. Feligioni M. (2019). Hyperhomocysteinemia as a risk factor and potential nutraceutical target for certain pathologies. Front. Nutr. 6, 49. 10.3389/fnut.2019.00049 31069230 PMC6491750

[B75] Tollis S. Rizzotto A. Pham N. T. Koivukoski S. Sivakumar A. Shave S. (2022). Chemical interrogation of nuclear size identifies compounds with cancer cell line-specific effects on migration and invasion. ACS Chem. Biol. 17 (3), 680–700. 10.1021/acschembio.2c00004 35199530 PMC8938924

[B76] Torrino S. Grasset E. M. Audebert S. Belhadj I. Lacoux C. Haynes M. (2021). Mechano-induced cell metabolism promotes microtubule glutamylation to force metastasis. Cell. Metab. 33, 13–135. 10.1016/j.cmet.2021.05.009 34102109

[B77] Tzounakas V. L. Anastasiadi A. T. Arvaniti V. Z. Lelli V. Fanelli G. Paronis E. C. (2022). Supplementation with uric and ascorbic acid protects stored red blood cells through enhancement of non-enzymatic antioxidant activity and metabolic rewiring. Redox Biol. 57, 102477. 10.1016/j.redox.2022.102477 36155342 PMC9513173

[B78] Van Loenhout J. Peeters M. Bogaerts A. Smits E. Deben C. (2020). Oxidative stress-inducing anticancer therapies: taking a closer look at their immunomodulating effects. Antioxidants 9, 1188. 10.3390/antiox9121188 33260826 PMC7759788

[B79] Wang Z. Liu F. Fan N. Zhou C. Li D. Macvicar T. (2020). Targeting glutaminolysis: new perspectives to understand cancer development and novel strategies for potential target therapies. Front. Oncol. 10, 589508. 10.3389/fonc.2020.589508 33194749 PMC7649373

[B80] Warburg O. (1956). On the origin of cancer cells. Science 123, 309–314. 10.1126/science.123.3191.309 13298683

[B81] Weber G. F. (2016). Metabolism in cancer metastasis. Int. J. Cancer 138, 2061–2066. 10.1002/ijc.29839 26355498

[B82] Wu H. Gong Y. Ji P. Xie Y. Jiang Y.-Z. Liu G. (2022). Targeting nucleotide metabolism: a promising approach to enhance cancer immunotherapy. J. Hematol. Oncol. 15, 45. 10.1186/s13045-022-01263-x 35477416 PMC9044757

[B83] Xue D. Zhou X. Qiu J. (2020). Emerging role of NRF2 in ROS-mediated tumor chemoresistance. Biomed. Pharmacother. 131, 110676. 10.1016/j.biopha.2020.110676 32858502

[B84] Yang H. Villani R. M. Wang H. Simpson M. J. Roberts M. S. Tang M. 2018. The role of cellular reactive oxygen species in cancer chemotherapy. J. Exp. Clin. Cancer Res. 37, 266. do 10.1186/s13046-018-0909-x 30382874 PMC6211502

[B85] Yu L. Zhang J. Li Y. (2022). Effects of microenvironment in osteosarcoma on chemoresistance and the promise of immunotherapy as an osteosarcoma therapeutic modality. Front. Immunol. 13, 871076. 10.3389/fimmu.2022.871076 36311748 PMC9608329

[B86] Zhang N. Xie T. Xian M. Wang Y.-J. Li H.-Y. Ying M.-D. 2016. SIRT1 promotes metastasis of human osteosarcoma cells. Oncotarget 7, 79654–79669. d 10.18632/oncotarget.12916 27793039 PMC5346743

[B87] Zhao Y. Zhao X. Chen V. Feng Y. Wang L. Croniger C. (2019). Colorectal cancers utilize glutamine as an anaplerotic substrate of the TCA cycle *in vivo* . Sci. Rep. 9, 19180. 10.1038/s41598-019-55718-2 31844152 PMC6915720

[B88] Zhuo B. Li Y. Li Z. Qin H. Sun Q. Zhang F. (2015). PI3K/Akt signaling mediated Hexokinase-2 expression inhibits cell apoptosis and promotes tumor growth in pediatric osteosarcoma. Biochem. Biophys. Res. Commun. 464, 401–406. 10.1016/j.bbrc.2015.06.092 26116768

